# Prediction model of poorly differentiated colorectal cancer (CRC) based on gut bacteria

**DOI:** 10.1186/s12866-022-02712-w

**Published:** 2022-12-20

**Authors:** Zhang Qi, Zuo Zhibo, Zhuang Jing, Qu Zhanbo, Han Shugao, Jin Weili, Liu Jiang, Han Shuwen

**Affiliations:** 1 Huzhou Central Hospital, Affiliated Central Hospital Huzhou University, No.1558, Sanhuan North Road, Wuxing District, Huzhou, Zhejiang Province 313000 People’s Republic of China; 2 First Hospital of Jiaxing, Jiaxing, Zhejiang Province People’s Republic of China; 3 Zhejiang Chinese Medical University, Hangzhou, Zhejiang Province People’s Republic of China; 4 Second Affiliated Hospital of School of Medicine, Zhejiang University, Hangzhou, Zhejiang Province People’s Republic of China; 5 Nanxun District People’s Hospital, Huzhou, Zhejiang Province People’s Republic of China; 6 Key Laboratory of Multiomics Research and Clinical Transformation of Digestive Cancer of Huzhou, Huzhou, People’s Republic of China

**Keywords:** Colorectal cancer (CRC), Poorly differentiated, Gut bacteria, Prediction model, Pathology

## Abstract

**Background:**

The mortality of colorectal cancer is high, the malignant degree of poorly differentiated colorectal cancer is high, and the prognosis is poor.

**Objective:**

To screen the characteristic intestinal microbiota of poorly differentiated intestinal cancer.

**Methods:**

Fecal samples were collected from 124 patients with moderately differentiated CRC and 123 patients with poorly differentiated CRC, and the bacterial 16S rRNA V1-V4 region of the fecal samples was sequenced. Alpha diversity analysis was performed on fecal samples to assess the diversity and abundance of flora. The RDP classifier Bayesian algorithm was used to analyze the community structure. Linear discriminant analysis and Student's t test were used to screen the differences in flora. The PICRUSt1 method was used to predict the bacterial function, and six machine learning models, including logistic regression, random forest, neural network, support vector machine, CatBoost and gradient boosting decision tree, were used to construct a prediction model for the poor differentiation of colorectal cancer.

**Results:**

There was no significant difference in fecal flora alpha diversity between moderately and poorly differentiated colorectal cancer (P > 0.05). The bacteria that accounted for a large proportion of patients with poorly differentiated and moderately differentiated colorectal cancer were *Blautia*, *Escherichia-Shigella*, *Streptococcus*, *Lactobacillus*, and *Bacteroides*. At the genus level, there were nine bacteria with high abundance in the poorly differentiated group, including *Bifidobacterium, norank_f__Oscillospiraceae, Eisenbergiella,* etc. There were six bacteria with high abundance in the moderately differentiated group, including *Megamonas, Erysipelotrichaceae_UCG-003, Actinomyces*, etc. The RF model had the highest prediction accuracy (100.00% correct). The bacteria that had the greatest variable importance in the model were *Pseudoramibacter, Megamonas* and *Bifidobacterium.*

**Conclusion:**

The degree of pathological differentiation of colorectal cancer was related to gut flora, and poorly differentiated colorectal cancer had some different bacterial flora, and intestinal bacteria can be used as biomarkers for predicting poorly differentiated CRC.

**Supplementary Information:**

The online version contains supplementary material available at 10.1186/s12866-022-02712-w.

## Introduction

Colorectal cancer (CRC) is the fourth most deadly cancer in the world, killing nearly 900,000 people each year. Advances in pathophysiological research have increased the number of treatment options for local and advanced disease, thereby facilitating the development of individual treatment regimens [1]. However, the clinicopathological features of poorly differentiated colorectal cancer have not been well studied due to the low frequency of occurrence of this type of cancer (3.3–18% of all colorectal cancer cases). The prognosis of patients with poorly differentiated colorectal cancer is generally reported to be worse and more adverse than that of patients with moderately differentiated cancer. The 5-year survival rate for poorly differentiated colorectal adenocarcinoma is 20% to 45.5% [2]. A large number of studies have shown that the occurrence and development of colorectal cancer are related to intestinal microbes. Therefore, a deeper understanding of the biology of poorly differentiated colorectal cancer can help analyze the biological changes of poorly differentiated tumors and determine the relationship between biology and pathology in colorectal cancer, thus providing a more theoretical basis for understanding the pathophysiology of poorly differentiated tumors. However, a way to bridge the gap between pathology and biology is needed.

In routine histopathological practice, tumor grade is one of the most important predictors of CRC aggressiveness [3]. The most widely accepted histopathological grading is based on tumor differentiation. When cancer is heterogeneous in differentiation, histopathological grading is determined according to the least differentiated components [4]. Compared with moderately differentiated tumors, poorly differentiated tumors have more solid structures. Poorly differentiated clusters (PDCs) are defined as ≥ 5 small populations of tumor cells without glandular differentiation, which has been considered a promising prognostic factor for colorectal cancer. A large number of studies have shown that poorly differentiated tumor morphology is significantly correlated with poor histopathological features and worse clinical prognosis [5]. In cytopathological studies, compared with moderately differentiated tumors, poorly differentiated tumors tend to have stronger invasiveness and growth, which is reflected in a stronger EMT and higher expression of cell proliferation capacity. The EMT is the process by which cells lose epithelial characteristics and acquire mesenchymal characteristics. In colorectal cancer, the EMT plays an important role in tumor progression, metastasis and drug resistance [6].

Mucosal epithelial cells are common targets of chronic bacterial infection and toxin damage, and most cancers originate from this tissue [7]. The gut microbiota is located near the colorectal epithelium and consists of a large number of microorganisms that interact with host cells to regulate many physiological processes, such as energy collection, metabolism and immune response, and sequencing studies have revealed microbial composition and ecological changes in CRC patients [8]. Some of these microbial characteristics have been used as biomarkers to improve the sensitivity of colorectal cancer diagnosis, and functional studies have shown the mechanism of certain bacteria in colorectal cancer [9]. Current studies have found that gut microbiota can affect circRNA expression to regulate the level of corresponding miRNAs, thus regulating the expression of genes related to the EMT [10]. In addition, some bacterial pathogens in the gut microbiome can exert tumor-promoting activity and interfere with important host cell signaling pathways related to cell proliferation by producing enzyme active protein toxins [11]. Therefore, imbalance of the gut microbiome is also associated with enhanced aggressiveness of poorly differentiated tumor cells.

The intestinal tract has an independent and complex microecosystem, and the interaction between different intestinal microbiota can maintain the homeostasis of the intestinal microenvironment, and jointly participate in the host's metabolism, material absorption and transformation process [12, 13]. Intestinal microorganisms are involved in the development of CRC [14]. The abundance of intestinal probiotics such as *Clostridium butyricum, Bifidobacterium, Lactobacillus* and *Bacteroides* decreased. The abundance of enterotoxin-producing *bacteroids fragilis, Escherichia coli, Clostridium difficile* and other pathogenic bacteria increased. Metagenomic analysis of stool samples from CRC patients has identified bacteria that are strongly associated with CRC development, including *Bacteroides fragilis*, *Fusobacterium nucleatum*, *Porphyromonas asaccharolytica*, *Parvimonas micra*, *Prevotella intermedia*, *Alistipes finegoldii* and *Thermanaerovibrio acidaminovorans* [15, 16]. Intestinal flora can play an anti-tumor role through its metabolites. For example, enterotoxins produced by *clostridium perfringens* can lead to the lysis of cancer cells through the imbalance of cell osmotic balance [17]. Ferritin secreted by *lactobacillus casei* directly induced apoptosis of tumor cells through the JNK pathway [18]. *Clostridium butyricum* can produce short-chain fatty acid sodium butyrate through anaerobic fermentation of dietary fiber [19]. The gut microbiota is complex, and there's a lot of research on CRC. However, there are few studies on the correlation between bacteria and the degree of pathological differentiation. We first reported that characteristic gut bacteria of poorly differentiated CRC were *Bifidobacterium, norank_f__Oscillospiraceae, Eisenbergiella*, etc., and the characteristic gut bacteria of moderately differentiated CRC were *Megamonas, Erysipelotrichaceae_UCG-003, Actinomyces*, etc. The relationship between the formation and transformation of different degrees of differentiation is unclear. It is difficult to confirm the relationship between microorganisms and different degrees of differentiation, and it needs a large number of animal and cell experiments to verify. The poor prognosis of poorly differentiated CRC has been clinically confirmed. It is of great significance to study the bacteria and the degree of differentiation to judge the different prognosis of the disease. Based on these characteristics, the prediction model was as a research method to screen important intestinal microorganisms. Screening characteristic microorganisms contribute to illustrate the mechanism of poorly and moderately differentiated CRC.

## Methods

### Subjects

The included subjects were 247 CRC patients (124 patients pathologically diagnosed with moderately differentiated CRC and 123 patients pathologically diagnosed with poorly differentiated CRC) at Huzhou Central Hospital from February 2019 to August 2020. Clinical staging followed the guidelines of the American Joint Council on Cancer (AJCC). The Huzhou Central Hospital ethics committee (No. 202202005–01) and Chinese clinical trial registry (http://www.chictr.org.cn, No. ChiCTR1800018908) approved the plan involving the patients’ clinical and informed consent. The raw sequencing data have been deposited into the NCBI Sequence Read Archive (SRA) database under the accession number of PRJNA904661 and PRJNA904946. The general situation of the patients is shown in Supplementary Table 1.

The inclusion criteria were moderately differentiated and poorly differentiated CRC confirmed by pathological examination.

The exclusion criteria were as follows: 1) complications with other malignant tumors; 2) serious heart and lung diseases; 3) oral history of gut bacteria preparation 1 month before admission; and 4) other intestinal diseases, such as ulcerative colitis and Crohn's disease.

### Fecal sample collection

Subjects were told to collect stool samples before breakfast. In the absence of laxatives or lubricants, approximately 5–10 g of stool samples were collected after defecation. Within half an hour, the samples were stored in a − 80° laboratory freezer. Fecal samples should not be stored for more than one month.

### MiSeq sequencing of the microbial genome

1. Genomic DNA extraction: A bacterial DNA extraction kit was used to extract bacterial DNA from fecal microbial samples, and a NanoDrop2000 was used for a DNA purity analysis. After selecting qualified samples, professional companies were commissioned to conduct 16S rRNA sequencing.
2. PCR amplification: Specific primers with barcodes were synthesized. The sequence of the 16S rDNA primer in the V1-V4 region was 357F: 5’-TACGGGAGGCAGCAG-3’; 1114R: 5’-GCAACGAGCGCAACCC-3’. To ensure the accuracy and reliability of subsequent data analysis, two conditions should be met: 1) amplification with a low cycle number should be used as much as possible; 2) the same number of amplification cycles should be ensured for each sample. A representative sample was randomly selected for the preexperiment to ensure that the majority of samples could be amplified at the appropriate concentration within the minimum number of cycles. Each sample had 3 replicates. PCR products of the same sample were mixed and detected by 2% agarose gel electrophoresis. An AxyPrepDNA Gel recovery Kit (AXYGEN company) was used to cut the gel and recover the PCR products, and Tris_HCl elution was performed with 2% agarose electrophoresis. The PCR products were detected and quantified using the QuantiFluor™-ST blue fluorescence quantification system (Promega company) based on the preliminary quantitative results of electrophoresis and then mixed in the appropriate proportion according to the sequencing volume requirements of each sample.
3. MiSeq library construction: We connected the "Y" shape connector, and magnetic beads were used to remove self-connecting segments. The library template was enriched by PCR amplification. Sodium hydroxide was denatured to produce single-stranded DNA fragments.
4. MiSeq sequencing: One end of the DNA fragment was complementary to the primer base and fixed on the chip; the other end was randomly complementary to another primer nearby, which was also fixed to form a "bridge." PCR amplification was used to produce DNA clusters, and the DNA amplicon then linearized into a single strand. The modified DNA polymerase and dNTPs with four fluorescent markers were added, and only one base was synthesized in each cycle. The surface of the reaction plate was scanned by laser, and the nucleotide species polymerized in the first reaction of each template sequence were read. The "fluorophore" and "terminator" were chemically cleaved to restore the viscosity of the 3' end and continued to polymerize the second nucleotide. The fluorescence signal collected in each round was counted, and the sequence of the template DNA fragment was obtained.

### Bioinformatics analysis

1. Data optimization and statistics: The double-ended sequence data required for MiSeq sequencing were first merged into a sequence according to the overlap relationship between PE reads, and the quality of reads and the effect of merging were controlled and filtered at the same time. The sequence direction was corrected according to the box sequence at the end of the sequence, and the samples were identified and differentiated according to the barcode label sequence to obtain effective data.
2. OTU cluster analysis: The UPARSE (Version 7.1) method was used to perform OTU clustering. The sequence similarity in OTUs was set to 97%, and the representative sequence of OTUs was obtained. Uchime (Version 4.2.40) was used to detect the chimeric sequences generated in PCR amplification and remove them from OTUs. The Usearch_global method was used to compare the optimized sequence map back to the OTU representative sequence and obtain the sequence abundance statistics table for each OTU sample.
3. Diversity analysis: Microbial diversity in fecal microbial community ecology was studied. The abundance and diversity of the microbial community can be reflected by diversity analysis of a single sample (alpha diversity), including a series of statistical analysis indices to estimate the species abundance and diversity of the environmental community. Mothur software (https://www.mothur.org/wiki/Download_mothur) was used to calculate the Chao abundance index and Ace index assessment flora. The Shannon index and Simpson index were calculated to evaluate the diversity of the bacterial community. GraphPad software was used to draw the violin diagram.
4. Community composition analysis: The RDP classifier Bayesian algorithm was used for taxonomic analysis of OTU representative sequences at a 97% similarity level, and the community composition of each sample was counted at the genus level. Variance decomposition was used to reflect the differences in multiple sets of data on the two-dimensional coordinate graph, and principal component analysis (PCA) was carried out by taking the two characteristic values that best reflected the variance values on the coordinate axis. The locations of samples in each dimension were recorded, and the contribution of each OTU to each principal component was calculated. PCA statistical analysis was conducted by using R language. PCoA analysis first sorted a series of feature values and feature vectors, then selected the most important feature values and presented them in the coordinate system. Then, R language was used for PCoA statistical analysis and mapping. A Venn diagram was used to analyze the number of species shared and unique by multiple samples in the moderately differentiated group and the poorly differentiated group to intuitively show the composition similarity and overlap of environmental samples at different classification levels (mostly at the OTU level).
5. Species difference analysis: According to the obtained community abundance data, Student's t test was used for analysis to detect the abundance differences of microbial communities in different groups (or samples) and to screen bacteria with significant differences. LEfSe multistage species difference discriminant analysis was performed. The nonparametric Kruskal–Wallis (KW) sum-rank test was used to detect significant differences in abundance, and the taxa with significant differences were identified. Finally, linear discriminant analysis (LDA) was used to estimate the impact of each component (species) abundance on the differential effect.
6. Correlation analysis: Student's t test and Tutools Platform software (http://www.cloudtutu.com), a free online data analysis website, was used to draw intragroup correlation heatmaps.
7. Functional prediction analysis: The PICRUSt1 method was used for the functional prediction of fecal sample flora genes. PICRUSt samples for expansion were sequenced, and the results predicted the function of the microbial community composition of the package (PICRUSt1 only for 16S sequencing data analysis function prediction, version 1.1.0 http://picrust.github.io/picrust/).

### Construction and validation of a prediction model for poorly differentiated colorectal cancer

To filter the differences of stool sample flora for building elements, we used an integrated application of logistic regression (LR), random forest (RF), neural network (NN), support vector machine (SVM) of CatBoost, and gradient boosted decision tree (GBDT) models. By including more decision tree classifiers, the results were determined on different decision trees, and the final classification was assigned after comprehensive consideration of all the results. For the results of classification problems, the probability maximum was taken, and the probability mean was taken for regression analysis to select the most important biomarker for sample classification. A series of sensitivity and specificity calculations were performed by setting different critical values for continuous variables. ROC curves were drawn with sensitivity as the ordinate and specificity as the abscissa, and the area under the curve (AUC) was calculated to build a prediction model for poor differentiation in colorectal patients. During data analysis, the data were divided into 70% training set to build the model and 30% test set to verify the model.

### Statistical analysis

For continuous variables, an independent t test was applied. For categorical variables between groups, Pearson’s chi-square test was used, depending on assumption validity. Statistical analysis was performed using SPSS V25.0 (SPSS Inc., Chicago, IL). GraphPad Prism version 8.0 (San Diego, CA) and the Tutools platform (http://www.cloudtutu.com) were used for the preparation of graphs. All tests of significance were two-sided, and *p*<0.05 or corrected *p*<0.05 was considered statistically significant.

## Results

### Analysis of the alpha diversity of bacteria from moderately and poorly differentiated CRC

**Fig. 1:**
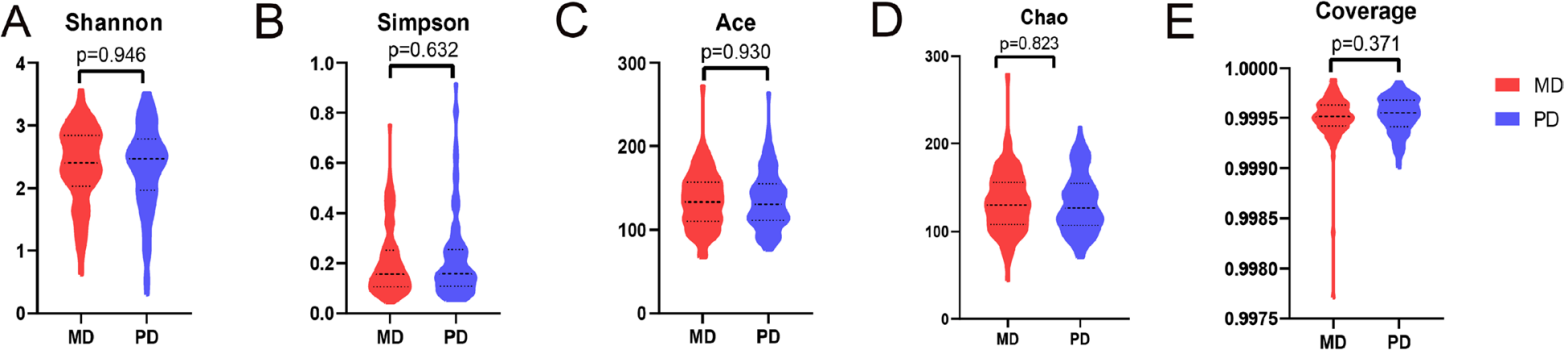
Alpha diversity analysis of the two groups with a violin diagram at the genus level. **A** Shannon index. **B** Simpson index. **C** Ace index. **D** Chao index. **E** Coverage index. * represents a significant difference between the two groups (*p* < 0.05)

The diversity of gut bacteria in patients with poor differentiation of CRC and those with moderate differentiation of CRC was compared, and there was no significant difference in the diversity of bacterial flora in stool samples between the two groups (*P* > 0.05), indicating that the diversity of intestinal bacterial flora in the two groups was basically the same (Fig. 1A-E, Supplementary Table 2.).

### Bacterial community structure of moderately and poorly differentiated CRC

**Fig. 2:**
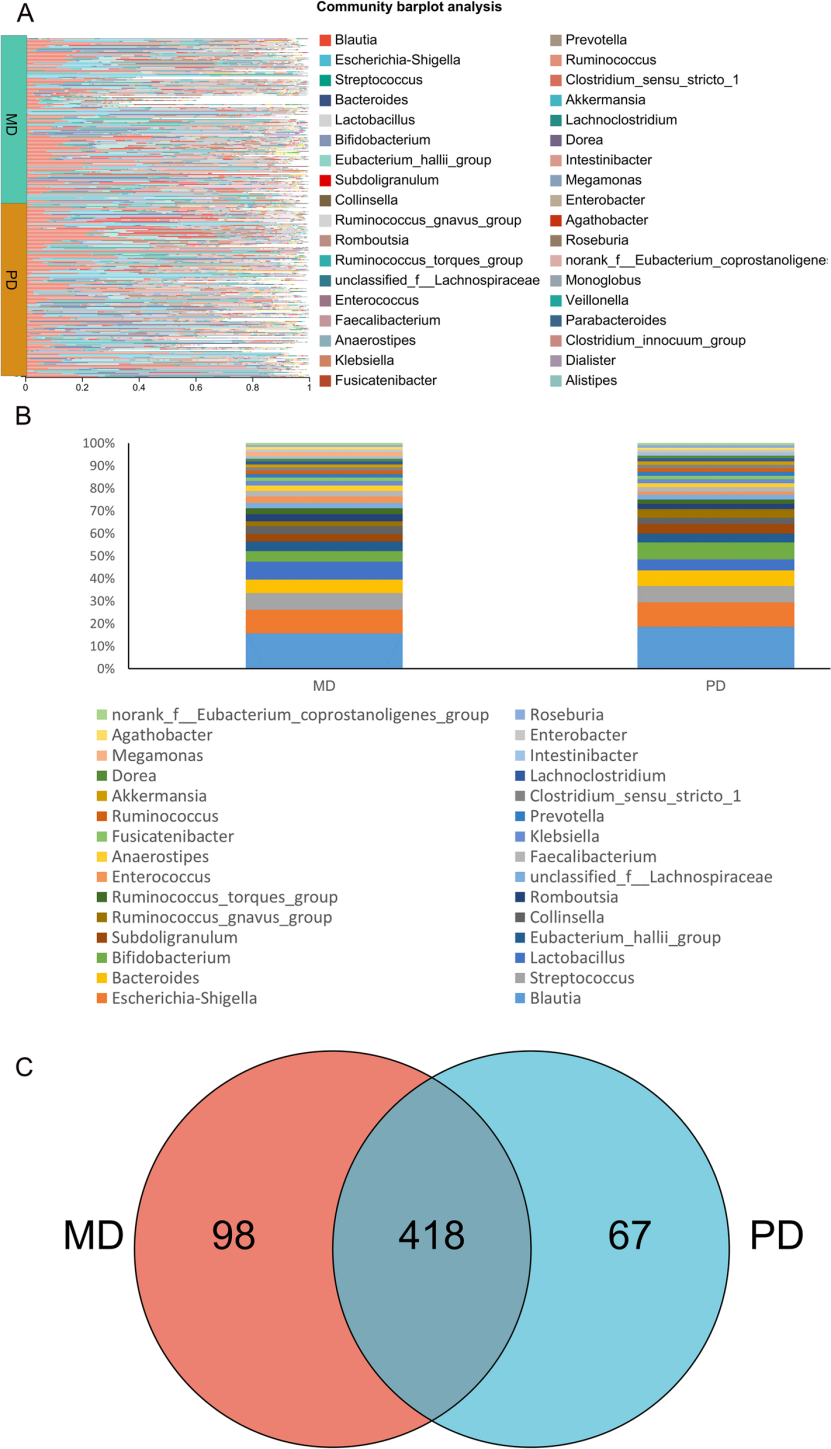
Composition of bacteria in stool samples from CRC patients. **A** Community composition of the intestinal bacterial community. The ordinate is the name of the sample, and the abscissa is the proportion of bacteria in the sample. Different colors of the column represent different species, and the length of the column represents the size of the proportion of the species. **B** A histogram of percentage accumulation drawn for the top 100 bacteria with the highest abundance in the two groups. **C** Venn diagram. Red represents the moderately differentiated group, blue represents the poorly differentiated group, and the number of nonoverlapping species represents the number of species unique to the corresponding group. Below is a Venn diagram of the total number of species in the two groups at the genus level

The community composition was analyzed to obtain the content of each genus at the level of each sample (Fig. 2A). The dominant species in each sample at the genus level mainly *included Blautia, Escherichia-Shigella,* and *Streptococcus.* The bacterial community structure in each sample was different, and the differences between samples were very large. The relative abundance of dominant species in the samples was different. By sequencing the top 30 bacteria in abundance and drawing the percentage accumulation histogram, it was found that the top five bacteria with high abundance in both groups were *Blautia, Escherichia-Shigella, Streptococcus, Lactobacillus* and *Bacteroides* (Fig. 2B). The number of common and unique OTUs among the two groups was counted, and there were 418 types of overlapping OTUs, including 98 types unique to CRC-differentiated groups and 67 types unique to CRC poorly differentiated groups (Fig. 2C).

### Differential gut bacteria between poorly and moderately differentiated CRC

**Fig. 3:**
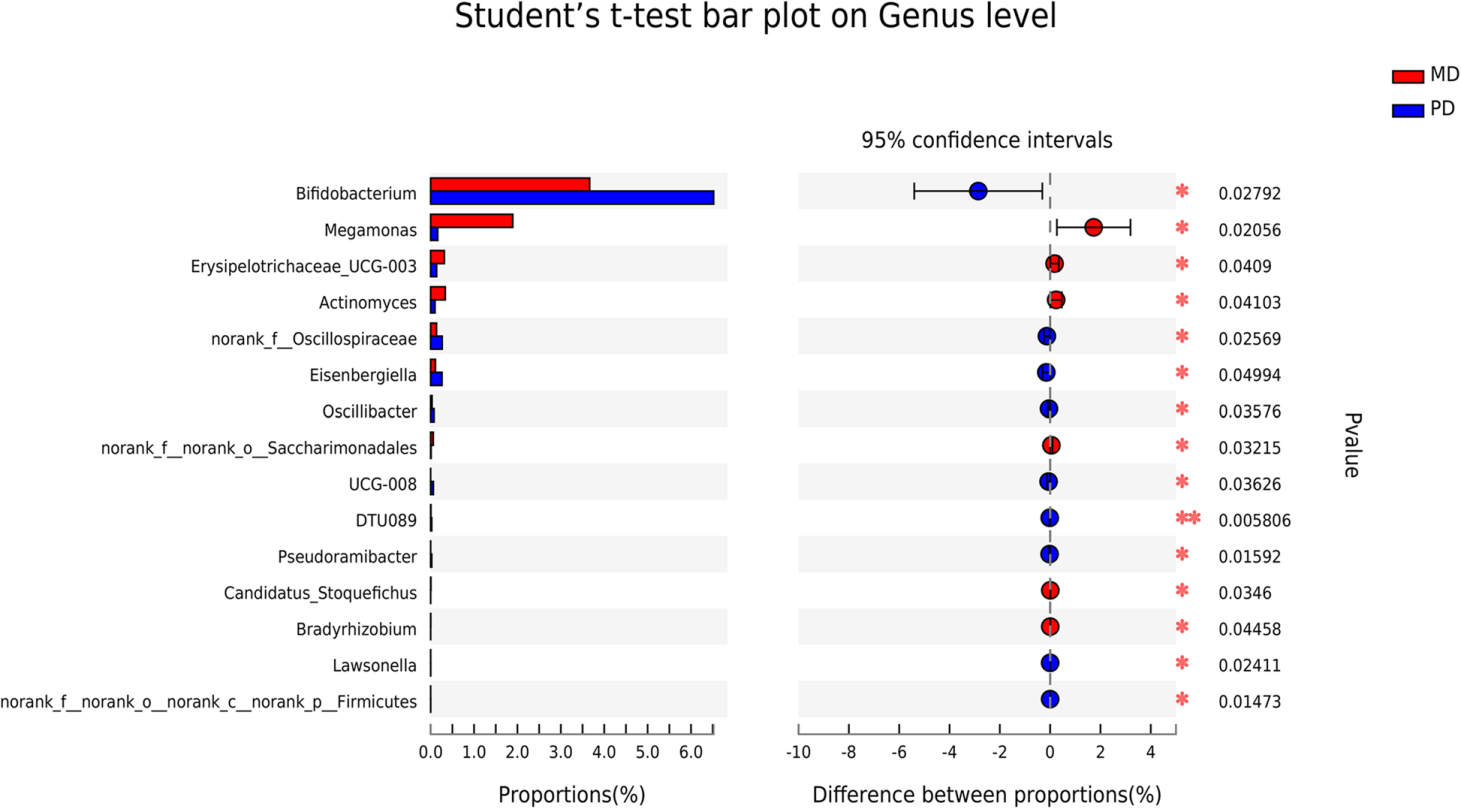
Multispecies difference test bar chart. Student's t test was used to test the hypothesis of species between the microbial communities of the two groups and evaluate the significance level of species abundance differences. *P* < 0.05 indicates a significant difference. The closer the line is to the middle, the smaller the standard deviation, and the better the central tendency

**Fig. 4:**
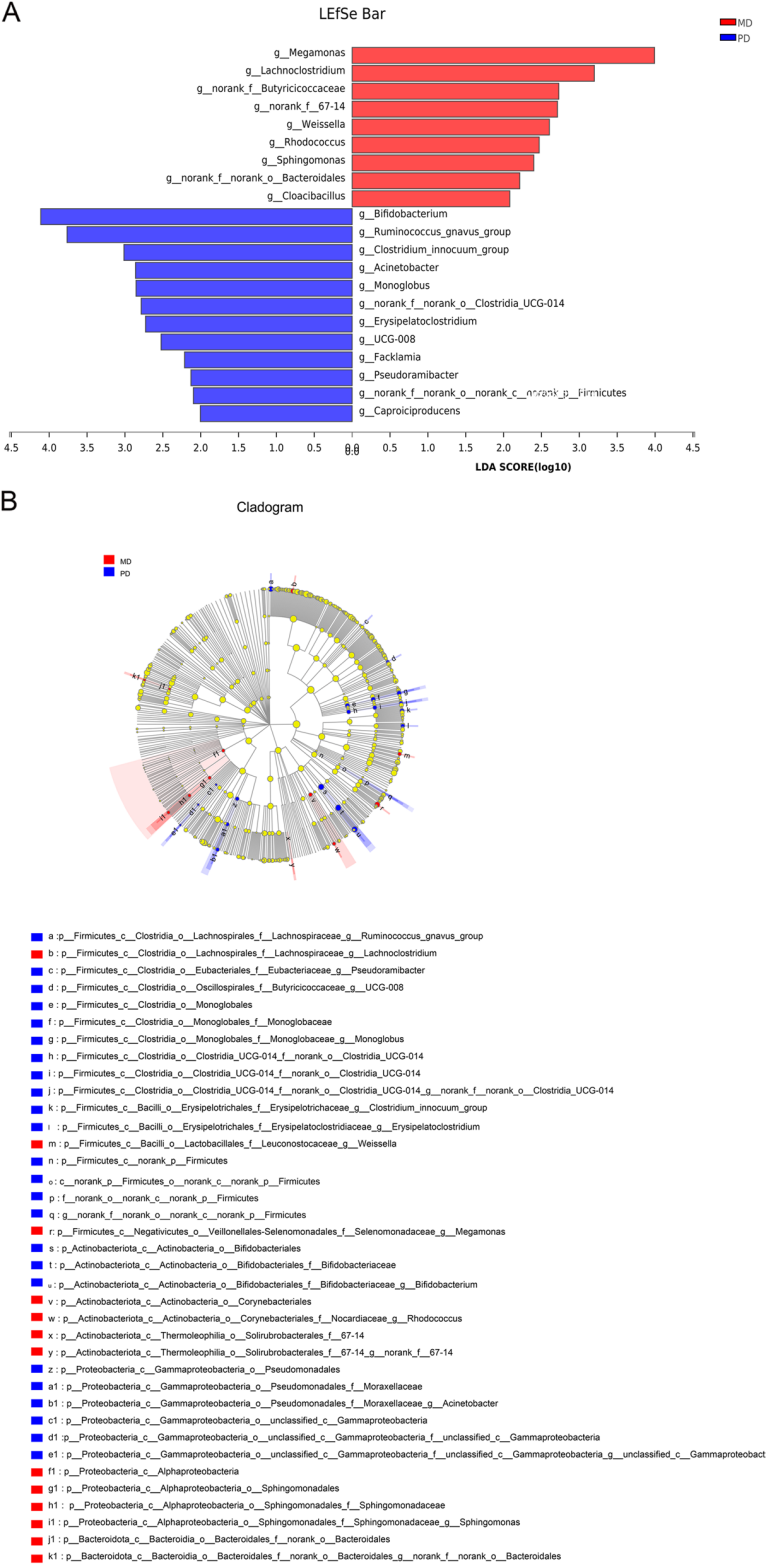
Diagram of different microflora between the two groups. **A** LDA was used to draw the histogram of LDA discriminant, and the microbial groups with significant effects in both groups were counted. The LDA score was obtained by linear regression analysis. The larger the LDA score, the greater the impact of bacterial abundance on the difference effect. LDA scores greater than 2 indicated statistically significant differences (*p* < 0.05). **B** The graph shows LEfSe multistage species from the inner to the outer circle and represents the phylum, class, order, family, genus, and species of different unit levels. Different color nodes indicate the microbial groups that were significantly enriched in the corresponding groups and had a significant influence on the differences between groups. The pale-yellow nodes indicate the microbial groups that had no significant difference among different groups or had no significant effect on the difference between groups

At the genus level, the differential bacteria between the patients with poorly differentiated CRC and the patients with moderately differentiated CRC were finally screened down to a total of 15 bacteria, At the genus level, there were nine bacteria with high abundance in the poorly differentiated group, including *Bifidobacterium, norank_f__Oscillospiraceae, Eisenbergiella,* etc. There were six bacteria with high abundance in the moderately differentiated group, including *Megamonas, Erysipelotrichaceae_UCG-003, Actinomyces*, etc. (Fig. 3). Finally, LEfSe LDA was used to estimate the impact of species abundance from the domain level to the genus level on each population. The bacteria that showed significant differences in the poorly differentiated CRC group were g_*Bifidobacterium, f_Bifidobacteriaceae, o_Bifidobacteriales*. The bacteria that showed significant differences in the moderately differentiated CRC group were g_*Megamonas,* g_*Lachnoclostridium,* g_*Corynebacteriales* (Fig. 4A-B).

### Correlation of differential bacteria

**Fig. 5:**
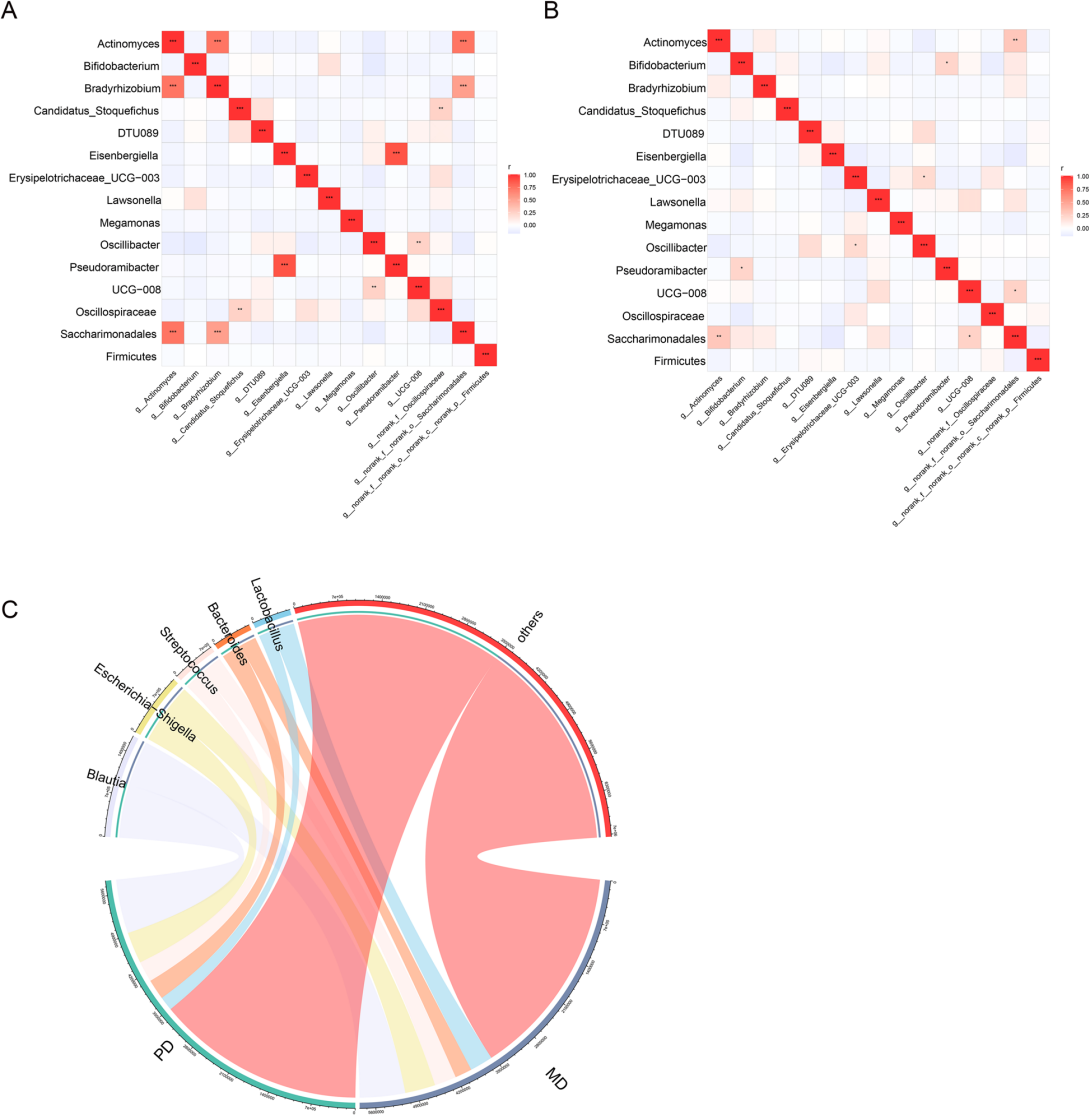
Correlation analysis of different bacteria within and between groups. The numerical matrix of the two groups of different bacteria is visually displayed through the heatmap. The color change reflects the data information, and the color depth represents the correlation. The redder the color, the higher the correlation between the two bacteria. **A** Intragroup bacterial correlation heatmap of the moderately differentiated CRC group. The Pearson coefficient was used to calculate the correlation between the bacteria. The shade of color indicates the size of the data value. Pearson correlation coefficients are indicated in the figure. * 0.01 < *p* < 0.05; * * 0.001 < *p* ≤ 0.01; * * * *p* ≤ 0.001. **B** Intragroup bacterial correlation heatmap of the poorly differentiated CRC group. **C** Chord diagram. One side of the circle is the species name, and the other side is the sample name, which is represented by different colors. The species abundance is displayed as a percentage

We analyzed the intragroup and intergroup correlation of different bacteria in the two groups. Figure 5A is a heatmap representing the correlation of bacteria in the moderately differentiated group. Figure 5B is a heatmap representing the correlation of bacteria in the poorly differentiated group. The network in the moderately differentiated group was different from that in the poorly differentiated group. Figure 5C is a chord diagram. Bacteria such as *Blautia, Escherichia − Shigella* and *Streptococcus* were abundant in both groups.

### Function prediction based on gut bacteria

**Fig. 6:**
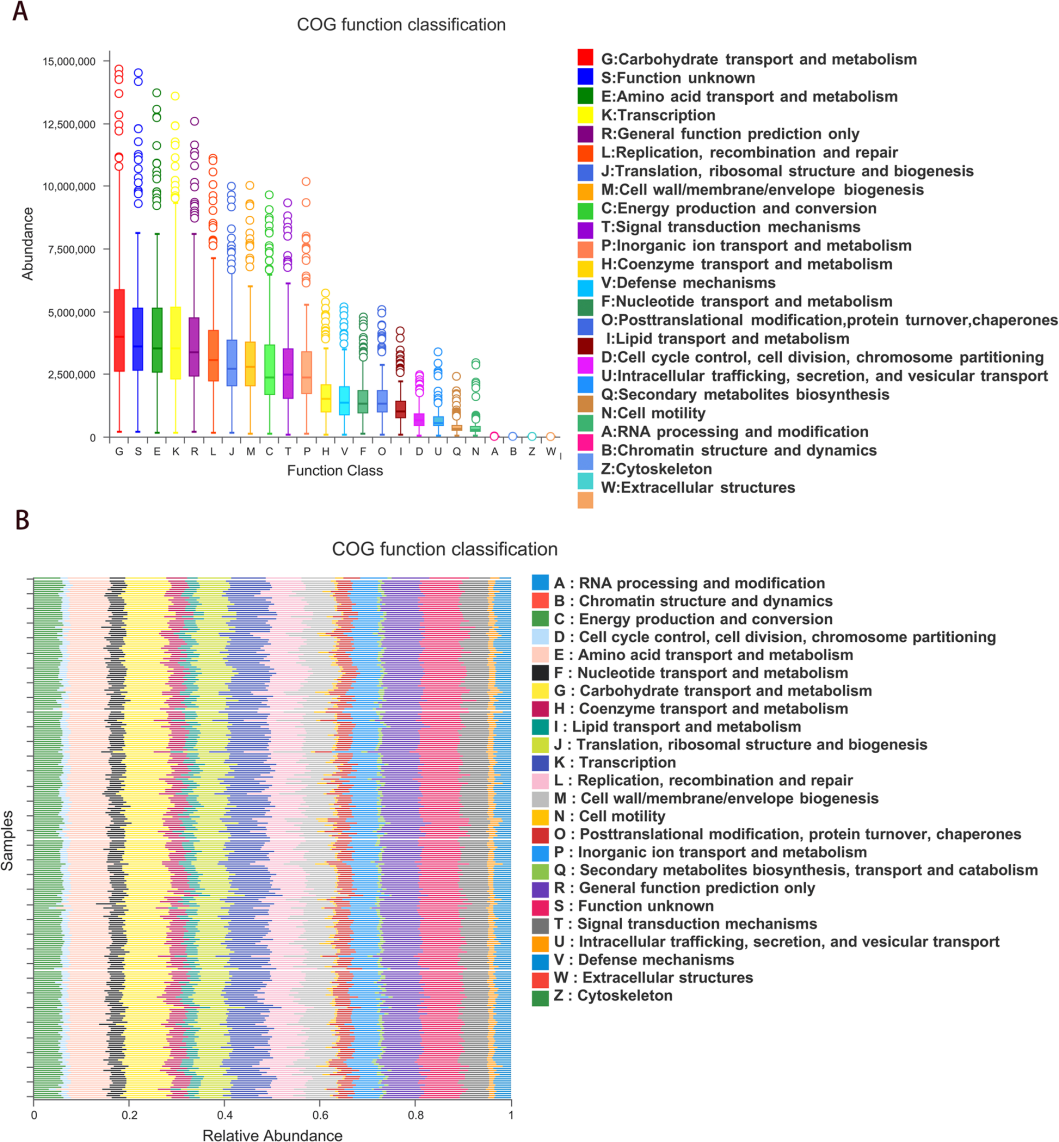
Diagram of the functional classification of bacteria. **A** Box diagram of COG functional classification. The abscissa represents the functional classification, and the ordinate represents the species abundance. **B** Histogram of COG functional classification. The abscissa represents the relative abundance, and the ordinate represents the groups

To further discuss the functions of intestinal bacteria, we analyzed the different functions of bacteria between the two groups. Figure 6A is the box diagram of COG functional classification. The abscissa represents the functional classification, and the ordinate represents the species abundance. Figure 6B is the histogram of COG functional classification. The two groups of different bacteria were enriched in 24 pathways, such as Carbohydrate transport and metabolism and amino acid transport and metabolism.

### Construction of a prediction model for poorly differentiated CRC

**Fig.7:**
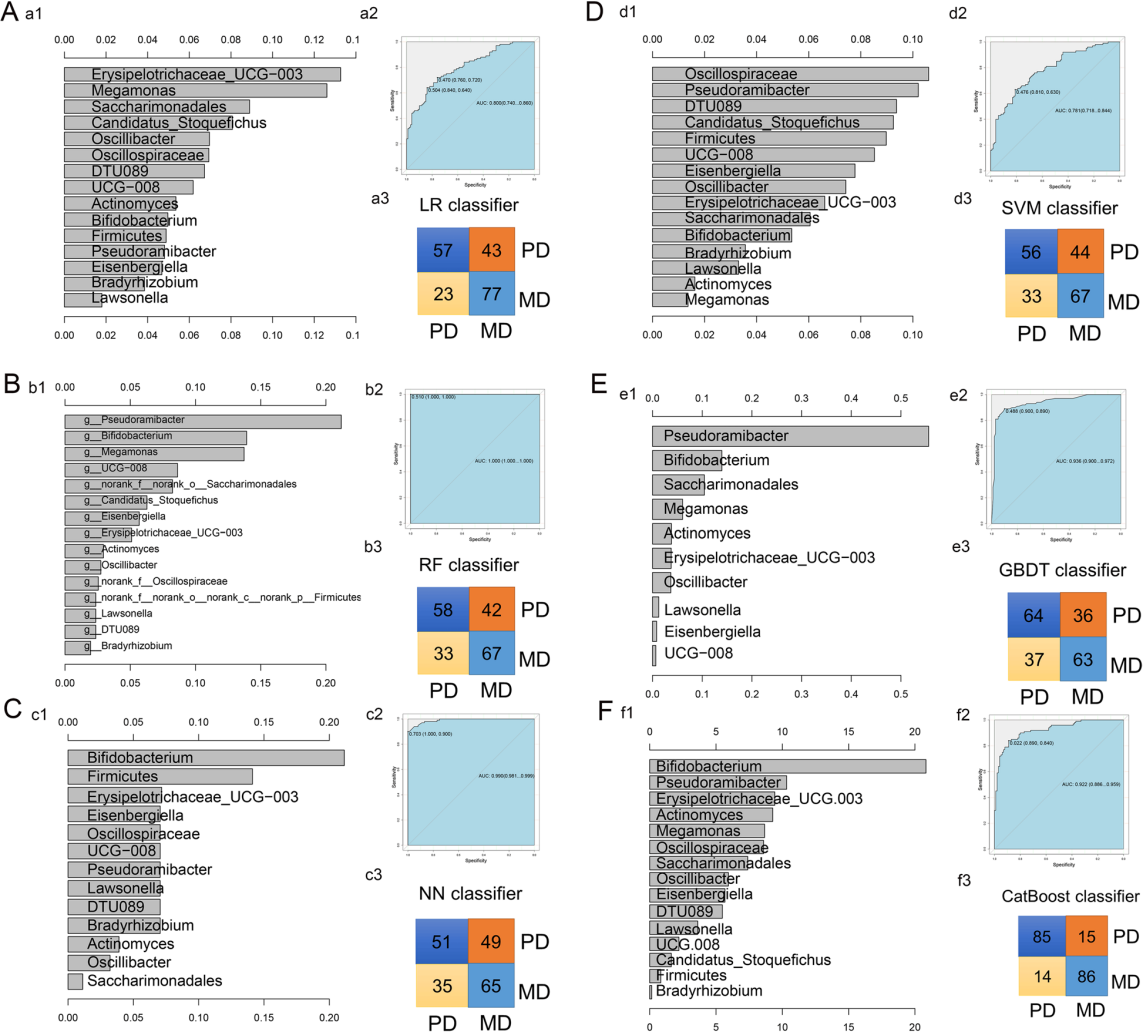
Construction of risk prediction model for poorly differentiated CRC. We used the relevant functions in the rminer Package (version 1.4.5) of R language for modeling analysis and used the fit function for modeling the variable importance calculation. **A-F** are the LR model (**A**), RF model (**B**), NN model (**C**), SVM model (**D**), GBDT model (**E**) and CatBoost model (**F**). The left panel (a1, b1, c1, d1, e1, f1) show the variable importance histogram of the model, the upper right panel (a2, b2, c2, d2, e2, f2) show the AUC curve of the model, and the lower right panel (a3, b3, c3, d3, e3, f3) show the CV. confuse matrix of the model

**Fig. 8:**
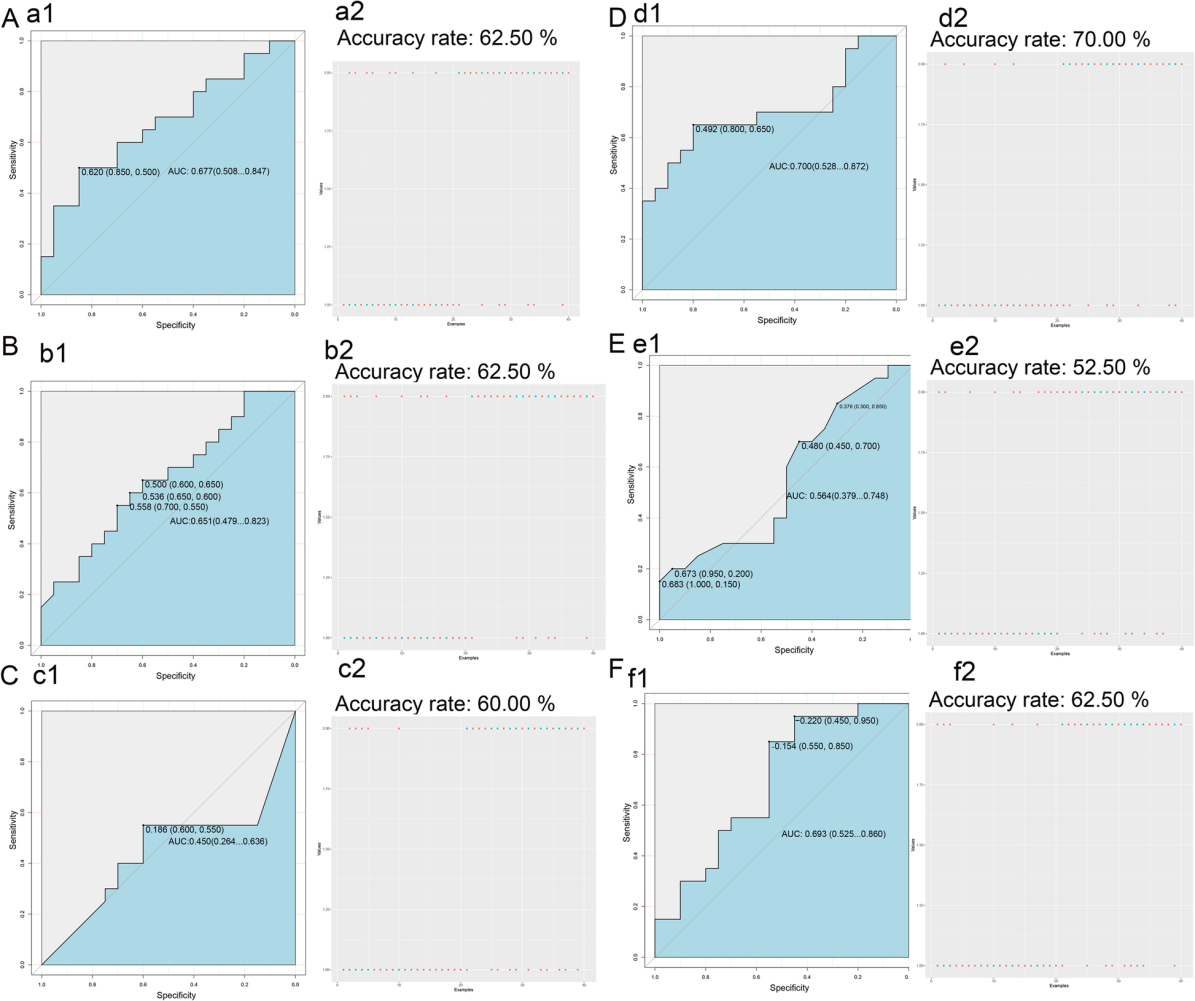
Validation of risk prediction model for poorly differentiated CRC. We used the relevant functions in the rminer Package (version 1.4.5) of R language for modeling analysis and used the fit function for modeling the variable importance calculation. All data were divided into 70% training set to build the model and 30% test set to verify the model. **A-F** are the LR model (**A**), RF model (**B**), NN model (**C**), SVM model (**D**), GBDT model (**E**) and CatBoost model (**F**). The left panel (a1, b1, c1, d1, e1, f1) show the AUC curve of the model, and the right panel (a2, b2, c2, d2, e2, f2) show the accuracy of the model

The LR, RF, NN, SVM, GBDT and CatBoost models were used to construct the prediction model of poor differentiation of CRC (Fig. 7A-F). Among the six models with different bacteria as factors, the RF model had the highest prediction accuracy (AUC = 1.00, 100.00% correct) (Fig. 7B). Among them*, Pseudoramibacter, Megamonas* and *Bifidobacterium* were the most important bacteria in the model. In model validation, the prediction accuracy of SVM model is the highest, the accuracy is 70.00%, and the value of AUC is 0.700 (Fig. 8A-F).

### The difference of poorly differentiated CRC and moderately differentiated CRC tissues

The histopathological features of poorly differentiated CRC were solid infiltrating pattern. (Supplementary Fig. 1A-B), and the histopathological features of moderately differentiated CRC were glandular pattern (Supplementary Fig. 1C-D).

### The age difference existed in the moderately and poorly differentiated groups

The results showed that the age difference existed in the moderately and poorly differentiated groups. Gut bacteria, such as *g_norank f_norank_o_Saccharimonadales* was related to age, *g_Megamonas* was related to sex, *g_DTU089* and *g_Pseudoramibacter* were related to differentiation degree.The age difference was shown in Supplementary Fig. 2.

## Discussion

The correlation between the microbiome and the occurrence and progression of CRC has received increasing attention, but determining how microbes influence cancer susceptibility and progression remains a challenge. Data from cross-sectional epidemiological studies, unbiased microbiome analyses of stool and colorectal tissue, and preclinical models reveal specific taxonomic and bacterial factors [20]. In addition, the role of gut flora in the host can also affect the degree of differentiation or malignancy of CRC in the process of cancer. Metagenomic analysis of stool samples from patients with CRC revealed bacteria closely associated with the development of CRC, and these bacteria included *Bacteroides fragilis, Fusobacterium nucleatum, Porphyromonas asaccharolytica, Parvimonas micra, Prevotella intermedia, Alistipes finegoldii,* and *Thermanaerovibrio acidaminovorans* [15, 16]. Molecular mechanisms that drive tumorigenesis have been elucidated, including bacterial membrane proteins or secretory molecules that interact with human cancer cells. However, for most gut bacteria, whether they enhance or inhibit the growth of cancer cells remains unknown [21]. At the same time, how gut bacterial disorders systematically affect the process and mechanism of the EMT in host CRC cells remains unclear. Abd-EI-Raouf et al. [22] studied the effect of bacteria on tumor cells after in vitro infection of bladder cancer cells by Escherichia coli and found that bacteria enhanced the EMT effect and improved the migration ability of cells. Studies have shown that Clostridium nucleatum can promote the EMT in oral squamous cell carcinoma by regulating the lncRNA MIR4435-2HG\/miR-296-5p\/Akt2\/SNAI1 signaling pathway, thus enhancing cell migration [23]. Lu et al. [24] proposed that the effector protein OspB, which is delivered by Shigella, affects cell proliferation by activating mTORC1, and that mTORC1 is the main regulator of cell growth. Studies have found that bifidobacteria may enhance the proliferation ability of colon epithelial cells by generating specific extracellular protein structure scaffolds to promote growth [25]. Therefore, whether gut bacteria can promote or inhibit the poor differentiation process of CRC and its possible mechanism are worth studying.

There have been many studies on the relationship between bacteria and tumors, and our research had similar findings. *Bifidobacterium* reduced cancer cell proliferation by inhibiting growth factor signaling and inducing mitochondria mediated apoptosis, and reduced chemical/immunological/radiotherapy side effects by inhibiting proinflammatory cytokines [26]. The research showed that the abundance of *Megamonas* in the gut microbiota of patients with cachexia was reduced, and the abundance of *Megamonas* was significantly different from the non-cachexia group, and it was possible that the disturbance of this microbiota was related to the later stage [27]. *Erysipelotrichaceae_UCG_003* was one of the main butyric acid-producing bacteria. The abundance of *Erysipelotrichaceae_UCG-003* in healthy group was significantly higher than that in lung cancer group, and it was negatively correlated with glycerol and phospholipid metabolism [28]. This negative correlation may be one of the ways to regulate metabolism and tumor development in vivo. In a research of the microbiota before and after chemotherapy, the abundance of *Actinomyces* in stool after chemotherapy increased to 2.5 times that before chemotherapy. *Actinomyces* in the gut may have a positive clinical outcome in CRC patients, and A*ctinomyces* may inhibit tumor growth [29]. *Oscillospiraceae* showed a relationship with metabolic disorders. In the intestinal microorganisms of patients with high uric acid, *Oscillospiraceae* was significantly reduced, and *Oscillospiraceae* may be related to uric acid metabolism [30]. However, there are few studies on the correlation between bacteria and the degree of pathological differentiation. We first reported that characteristic gut bacteria of poorly differentiated CRC were *Bifidobacterium, norank_f__Os*cillospiraceae, *Eisenbergiella*, etc., and the characteristic gut bacteria of moderately differentiated CRC were *Megamonas*, *Erysipelotrichaceae_UCG-003*, *Actinomyces*, etc. At present, the differentiation degree of CRC is rarely analyzed with gut bacteria, and poor differentiation is an important manifestation of more invasive CRC. In the present study, certain clinical samples were included to screen and analyze the bacterial characteristics of CRC tumor differentiation. The differences in intestinal microbiota from the perspective of pathological characteristics of CRC were explored, and the relationship between intestinal microbiota and biological behavior of CRC were studied. The study will provide a direction for the further research with strong innovations.

The association between poor differentiation of CRC and gut bacteria was significant. Dysbiosis of gut microbiota and subsequent inappropriate immune responses can lead to susceptibility to chronic inflammation, which contributes to the development of disease and cancer. Microorganisms may contribute to genetic and epigenetic changes through the production of superoxide radicals and genotoxins, as well as toll-like receptor-mediated oncogenic pathway induction [31]. The structural changes of gut bacteria and CRC differentiation may be mutually reinforcing. The increase in the number of specific microorganisms and the decrease in beneficial bacteria may increase the risk of poor differentiation of CRC and promote more invasive CRC. In addition, the poor differentiation state of CRC may also interfere with the intestinal microbiota structure, induce intestinal microbiota disorder, and further increase the probability of malignancy. Recently, *Helicobacter hepaticus* was found to increase tumor invasion by cytotoxic lymphocytes in mouse models, and this method can inhibit tumor growth [32]. This indicated that the differential microorganisms screened out in this study can become potential targets for the prevention or treatment of poor differentiation of CRC.

In addition, it was found that bacteria were correlated with each other through an intragroup correlation. Therefore, it is possible that different bacteria may participate in or assist each other in promoting or inhibiting the poor differentiation of CRC. In the follow-up study, microbial sequencing of poorly differentiated CRC is likely to find that the bacteria are consistent with those found in this study.

We further constructed a prediction model for poor differentiation of CRC based on differential gut bacteria, The research screened out important characteristic gut microbes, including *Pseudoramibacter, Megamonas* and *Bifidobacterium*. Liao et al. [33] established a kNN classification model based on the clinicopathological information and protein expression profile analysis of CRC, which predicted the degree of tumor differentiation of CRC with high accuracy (*P* ≤ 0.001, receiver-operator characteristics-ROC-error, 0.171). The expression of related genes, such as HER3 and insulin receptor substrate 1, was found to be a predictive target of the degree of CRC differentiation [34, 35]. Metabolites from gut microbes also play an important role. Symbiotic microbial factors are short-chain fatty acids, such as butyrate, which reduce the growth of normal intestinal stem cells and a range of cancer-derived cell lines [36]. The potential role of gut bacterial metabolites in CRC differentiation was not considered in this study. This may be one of the problems of this research experiment. At the same time, gut bacteria are affected by dietary habits, drug use and other factors, so the impact on the results of this analysis is inevitable. In the present study, the MiSeq platform was used for the second-generation sequencing of 16S rRNA V1-V4 region. Our research design began in 2018, and the third-generation sequencing technologies, such as NanoPore, were immature at that time. Third-generation sequencing can make the length of sequencing up to about 10 kb, and do not need PCR enrichment sequence, can be directly sequenced, third-generation sequencing can solve the problem of information loss and base mismatch. Long-read platforms will provide us with a direction in the further research. Vuik et al. [37] found that poorly differentiated CRCs were more common in the younger group by recruiting 6400 subjects. Pereira et al. [38] analyzed the pathological features of CRC in different groups of age and showed that poorly differentiated tumors was most common in young CRC patients. The results showed that the age difference existed in the moderately and poorly differentiated groups. In future studies, the age-related differential bacteria could be further analyzed. At the same time, the sample size of this study is insufficient, which also limits the applicability of the research results. In the future, multicenter studies are needed to further verify whether these microbiota can be used as promoting factors for the development of CRC, and to further find the link between these microbiota and the development of CRC.

## Supplementary Information

**Additional file 1: Supplementary Figure 1.** 

**Additional file 2: Supplementary Figure 2.** 

**Additional file 3: Supplementary Table  1.** Clinical information on patients with moderately andpoorly differentiated colorectal cancer.

**Additional file 4: Supplementary Table 2.** Table of diversity indices. The indexes of community richness werechao and ace. The indexes of community diversity were shannon, simpson and coverage.

## Data Availability

The datasets generated for this study can be accessed from the NCBI Sequence Read Archive (SRA) database under the accession number PRJNA904661 (http://www.ncbi.nlm.nih.gov/bioproject/904661) and PRJNA904946 (http://www.ncbi.nlm.nih.gov/bioproject/904946). The data has been released to the public.

## Abbreviations

EMT: Epithelial mesenchymal transformation
LDA: Linear discriminant analysis
LR: Logistic regression
RF: Random forest
NN: Neural network
GBDT: Gradient boosted decision tree
SVM: Support vector machine
kNN: K-Nearest Neighbor

## References

[1] Dekker E, Tanis PJ, Vleugels JLA (2019). Colorectal cancer. Lancet.

[2] Komori K, Kanemitsu Y, Ishiguro S (2011). Clinicopathological study of poorly differentiated colorectal adenocarcinomas: comparison between solid-type and non-solid-type adenocarcinomas. Anticancer Res.

[3] Reggiani Bonetti L, Barresi V, Bettelli S (2016). Poorly differentiated clusters (PDC) in colorectal cancer: what is and ought to be known. Diagn Pathol.

[4] Tajima Y, Shimada Y, Kameyama H (2017). Association between poorly differentiated clusters and efficacy of 5-fluorouracil-based adjuvant chemotherapy in stage III colorectal cancer. Jpn J Clin Oncol.

[5] Shivji S, Conner JR, Barresi V (2020). Poorly differentiated clusters in colorectal cancer: a current review and implications for future practice. Histopathology.

[6] Zhang N, Ng AS, Cai S (2021). Novel therapeutic strategies: targeting epithelial-mesenchymal transition in colorectal cancer. Lancet Oncol.

[7] Dziubanska-Kusibab PJ, Berger H, Battistini F (2020). Colibactin DNA-damage signature indicates mutational impact in colorectal cancer. Nat Med.

[8] Wong SH, Yu J (2019). Gut microbiota in colorectal cancer: mechanisms of action and clinical applications. Nat Rev Gastroenterol Hepatol.

[9] Kwong TNY, Wang X, Nakatsu G (2018). Association Between Bacteremia From Specific Microbes and Subsequent Diagnosis of Colorectal Cancer. Gastroenterology.

[10] Zhu Z, Huang J, Li X (2020). Gut microbiota regulate tumor metastasis via circRNA/miRNA networks. Gut Microbes.

[11] Fiorentini C, Carlini F, Germinario EAP (2020). Gut Microbiota and Colon Cancer: A Role for Bacterial Protein Toxins?. Int J Mol Sci.

[12] Tilg H, Adolph TE, Gerner RR (2018). The Intestinal Microbiota in Colorectal Cancer[J]. Cancer Cell.

[13] Shen ZH, Zhu CX, Quan YS (2018). Relationship between intestinal microbiota and ulcerative colitis: Mechanisms and clinical application of probiotics and fecal microbiota transplantation[J]. World J Gastroenterol.

[14] Fan X, Jin Y, Chen G (2021). Gut microbiota dysbiosis drives the development of colorectal cancer. Digestion.

[15] Dai Z, Coker OO, Nakatsu G (2018). Multi-Cohort analysis of colorectal cancer metagenome identified altered bacteria across populations and universal bacterial markers[J]. Microbiome.

[16] Wirbel J, Pyl PT, Kartal E (2019). Meta-Analysis of fecal metagenomes reveals global microbial signatures that are specific for colorectal cancer[J]. Nat Med.

[17] Vecchio AJ, Rathnayake SS, Stroud RM (2021). Structural basis for Clostridium perfring ens enterotoxin targeting of claudins at tight junctions in mammalian gut. Proc Natl Acad Sci U S A..

[18] Konishi H, Fujiya M, Tanaka H (2016). Probiotic-derived ferrichrome inhibits colon cancer progression via JNK-mediated apoptosis. Nat Commun.

[19] Stoeva MK, Garcia-So J, Justice N (2021). Butyrate-producing human gut symbiont, Clostridium butyricum, and its role in health and disease. Gut Microbes.

[20] Clay SL, Fonseca-Pereira D, Garrett WS (2022). Colorectal cancer: the facts in the case of the microbiota. J Clin Invest.

[21] Taddese R, Garza DR, Ruiter LN (2020). Growth rate alterations of human colorectal cancer cells by 157 gut bacteria. Gut Microbes.

[22] Abd-El-Raouf R, Ouf SA, Gabr MM (2020). Escherichia coli foster bladder cancer cell line progression via epithelial mesenchymal transition, stemness and metabolic reprogramming. Sci Rep.

[23] Zhang S, Li C, Liu J (2020). Fusobacterium nucleatum promotes epithelial-mesenchymal transiton through regulation of the lncRNA MIR4435-2HG/miR-296-5p/Akt2/SNAI1 signaling pathway. FEBS J.

[24] Lu R, Herrera BB, Eshleman HD (2015). Shigella Effector OspB Activates mTORC1 in a Manner That Depends on IQGAP1 and Promotes Cell Proliferation. PLoS Pathog.

[25] O'Connell Motherway M, Houston A, O'Callaghan G (2019). A Bifidobacterial pilus-associated protein promotes colonic epithelial proliferation. Mol Microbiol.

[26] Badgeley A, Anwar H, Modi K (2021). Effect of probiotics and gut microbiota on anti-cancer drugs: Mechanistic perspectives. Biochim Biophys Acta Rev Cancer.

[27] Ubachs J, Ziemons J, Soons Z (2021). Gut microbiota and short-chain fatty acid alterations in cachectic cancer patients. J Cachexia Sarcopenia Muscle.

[28] Zhao F, An R, Wang LQ (2021). Specific Gut Microbiome and Serum Metabolome Changes in Lung Cancer Patients. Front Cell Infect Microbiol.

[29] Li J, Chu RX, Wang CZ (2020). Microbiome characteristics and Bifidobacterium longum in colorectal cancer patients pre- and post-chemotherapy. Transl Cancer Res.

[30] Zhang WF, Wang T, Guo RX (2022). Variation of Serum Uric Acid Is Associated With Gut Microbiota in Patients With Diabetes Mellitus. Front Cell Infect Microbiol.

[31] DeDecker L, Coppedge B, Avelar-Barragan J (2021). Microbiome distinctions between the CRC carcinogenic pathways. Gut Microbes.

[32] Overacre-Delgoffe AE, Bumgarner HJ, Cillo AR (2021). Microbiota-specific T follicular helper cells drive tertiary lymphoid structures and anti-tumor immunity against colorectal cancer. Immunity.

[33] Liao CC, Ward N, Marsh S (2010). Mass spectrometry protein expression profiles in colorectal cancer tissue associated with clinico-pathological features of disease. BMC Cancer.

[34] Nakata S, Tanaka H, Ito Y (2014). Deficient HER3 expression in poorly-differentiated colorectal cancer cells enhances gefitinib sensitivity. Int J Oncol.

[35] Lomperta K, Jakubowska K, Grudzinska M (2020). Insulin receptor substrate 1 may play divergent roles in human colorectal cancer development and progression. World J Gastroenterol.

[36] Bell HN, Rebernick RJ, Goyert J (2022). Reuterin in the healthy gut microbiome suppresses colorectal cancer growth through altering redox balance. Cancer Cell.

[37] Vuik FER, Nieuwenburg SAV, Nagtegaal ID (2021). Clinicopathological characteristics of early onset colorectal cancer. Aliment Pharmacol Ther.

[38] Pereira AAL, Fernandes GDS, Braga GTP (2020). Differences in Pathology and Mutation Status Among Colorectal Cancer Patients Younger Than, Older Than, and of Screening Age. Clin Colorectal Cancer.

