# Supplementary figures and images for: Prediction model of poorly differentiated colorectal cancer (CRC) based on gut bacteria
Source: BMC Microbiol. 2022 Dec 20;22:312. doi: 10.1186/s12866-022-02712-w (PMC9764708; doi:10.1186/s12866-022-02712-w)

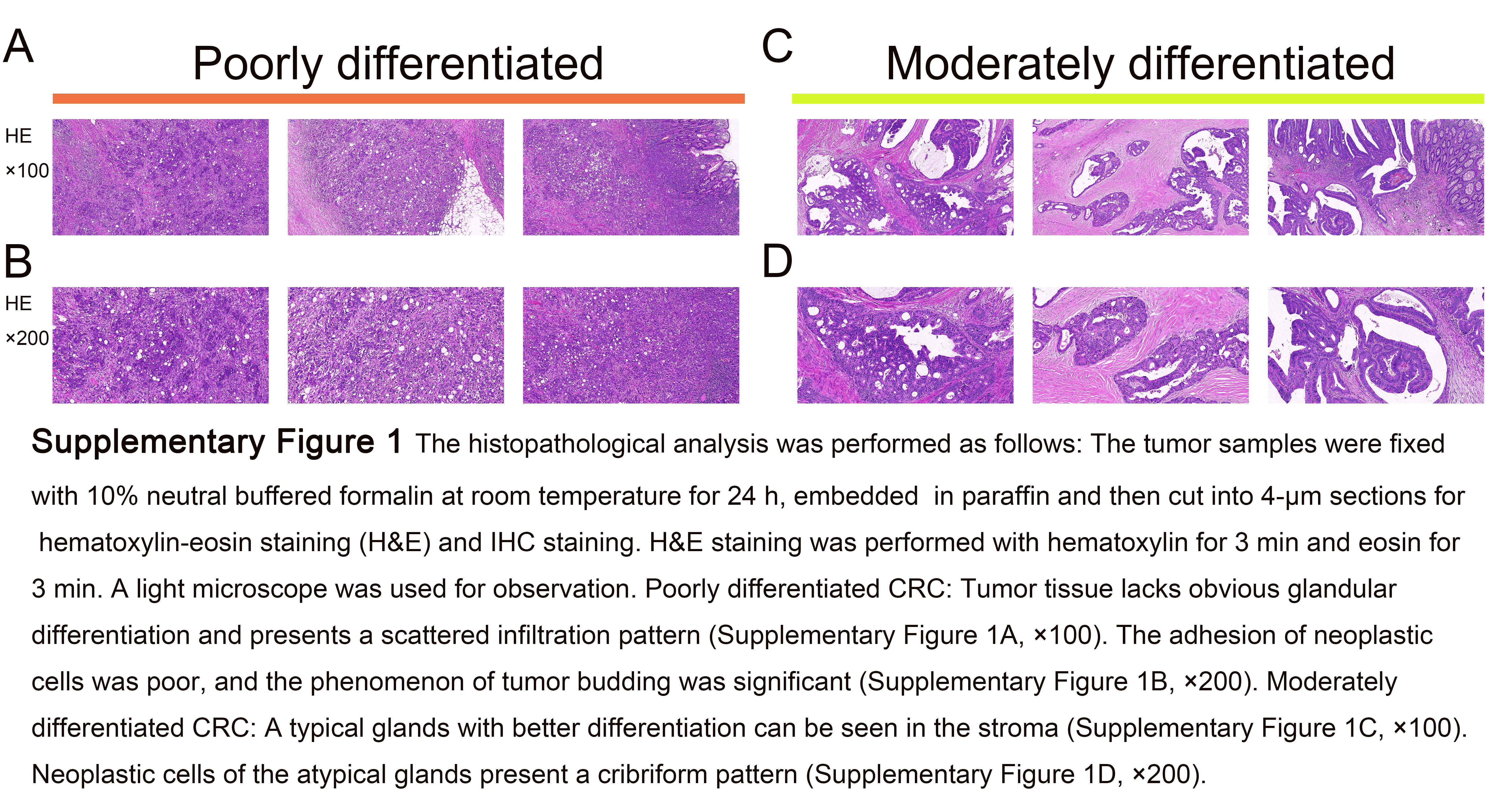

Supplement: Supplementary file 1 — Additional file 1: Supplementary Figure 1. [file 12866_2022_2712_MOESM1_ESM.gif]

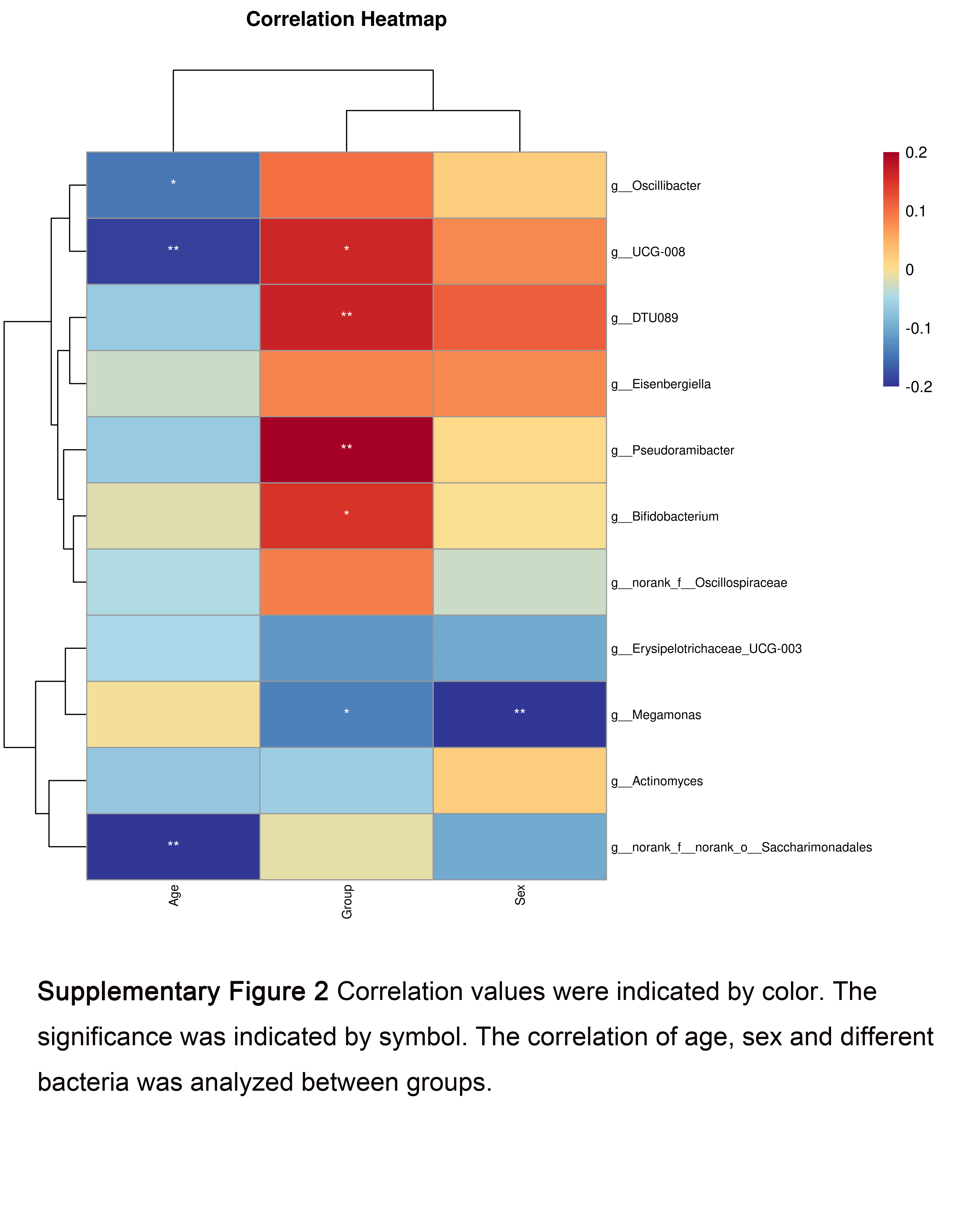

Supplement: Supplementary file 2 — Additional file 2: Supplementary Figure 2. [file 12866_2022_2712_MOESM2_ESM.gif]
