# Supplementary material for: Prediction model of poorly differentiated colorectal cancer (CRC) based on gut bacteria
Source: BMC Microbiol. 2022 Dec 20;22:312. doi: 10.1186/s12866-022-02712-w (PMC9764708; doi:10.1186/s12866-022-02712-w)
Supplement: Supplementary file 3 — Additional file 3: Supplementary Table 1. Clinical information on patients with moderately andpoorly differentiated colorectal cancer. [file 12866_2022_2712_MOESM3_ESM.doc]

Supplementary Table 1 Clinical information on patients with moderately and poorly differentiated colorectal cancer

|  |  | | Moderately differentiated CRC group | Poorly differentiated CRC group | P- value |
| --- | --- | --- | --- | --- | --- |
| Cases,n |  | 124 | | 123 |  |
| Sex | Male,n | 92 | | 76 | 0.010 |
|  | Female,n | 32 | | 47 |  |
| Age |  | 66.93±24.09 | | 61.76±9.79 | 0.028 |
| BMI(kg/m^2^) |  | 22.73±3.23 | | 23.04±4.04 | 0.510 |
| Total protein(g/L) |  | 68.41±5.76 | | 70.57±7.91 | 0.033 |
| GOT(U/L) |  | 24.38±11.29 | | 28.17±15.82 | 0.040 |
| Cr(μmol/L) |  | 74.8±16.38 | | 70.76±15.24 | 0.055 |
| RBC(10^12/L) |  | 4.42±4.36 | | 4.04±0.53 | 0.357 |
| Hb(g/L) |  | 125.96±18.53 | | 123.76±20.13 | 0.392 |
| WBC(10^9/L) |  | 5.11±2.18 | | 5.33±1.86 | 0.428 |
| Blood platelet(10^9/L) |  | 172.53±67.35 | | 187.71±74.71 | 0.109 |
| Albumin（g/L） |  | 39.37±5.46 | | 40.16±4.82 | 0.258 |
| TG(mmol/L) |  | 1.66±1 | | 1.41±0.76 | 0.056 |
| TC(mmol/L) |  | 4.52±0.91 | | 4.65±1 | 0.368 |
| HDL(mmol/L) |  | 47.33±13.25 | | 48.02±12.86 | 0.721 |
| LDL(mmol/L) |  | 95.69±31.31 | | 104.66±34.13 | 0.067 |
| Lp-a(mmol/L) |  | 24.36±28.28 | | 35.89±38.66 | 0.023 |
