# Supplementary material for: Prediction model of poorly differentiated colorectal cancer (CRC) based on gut bacteria
Source: BMC Microbiol. 2022 Dec 20;22:312. doi: 10.1186/s12866-022-02712-w (PMC9764708; doi:10.1186/s12866-022-02712-w)
Supplement: Supplementary file 4 — Additional file 4: Supplementary Table 2. Table of diversity indices. The indexes of community richness werechao and ace. The indexes of community diversity were shannon, simpson and coverage. [file 12866_2022_2712_MOESM4_ESM.pdf]

Supplementary Table 2. Table of diversity indices. The indexes of community richness were chao and ace. The indexes of community diversity were shannon, simpson and coverage.

Groups	shannon	simpson	ace	chao	coverage
	3.166782	0.089623	164.3581	162.0667	0.999661
	1.704558	0.431855	104.7626	108.6667	0.999506
	2.359165	0.189272	146.8516	143	0.999655
	2.20945	0.24796	145.7755	138.1429	0.999565
	1.61447	0.362149	161.7642	149.1364	0.999507
	3.117144	0.0746	162.9399	163.1538	0.999696
	3.567587	0.042333	191.2692	194	0.999655
	2.923145	0.13067	187.341	200.2727	0.999414
	2.023625	0.263997	98.44888	107.6667	0.99953
	2.30614	0.190802	113.1434	106.3636	0.999783
	2.502782	0.164385	106.2607	111.4286	0.999627
	2.966804	0.088518	136.1243	134.9286	0.999411
	2.789407	0.117462	174.5545	169	0.999377
	1.002611	0.445429	91.05357	90.3	0.999779
	3.204026	0.067651	180.3732	178.1176	0.999434
	2.277931	0.19025	146.7061	143.2143	0.999313
	2.874628	0.145777	156.5198	152.125	0.999474
	1.969087	0.355755	132.9695	121.6667	0.999455
	1.316525	0.357976	96.72867	100.8571	0.999781
	2.212015	0.198261	119.3357	119	0.999581
	2.317737	0.223616	132.1027	127.8333	0.999669
	2.511856	0.138708	151.061	183	0.999291
	3.001321	0.090852	181.3922	173.1111	0.999385
	1.017588	0.441035	89.31935	80	0.999404
	2.548312	0.208761	127.8078	128.6667	0.999564
	1.347408	0.543971	140.689	139	0.999452
	1.476564	0.49732	272.0951	279.6154	0.998361
	0.614937	0.753332	128.4493	84	0.999581
	2.881679	0.101294	152.2741	145.5	0.999441
	1.814241	0.276833	154.6916	133.5	0.99938
	3.129163	0.070969	155.282	153	0.999401
	2.871959	0.094366	125.0823	124.1	0.999558
	1.233087	0.49731	135.7576	128.5	0.99929
	2.701831	0.153321	137.3416	140.1	0.999497
	1.372269	0.353873	99.51259	78	0.999811
	2.518701	0.120825	118.9863	114.6875	0.999871
	2.233684	0.190388	135.3505	133.25	0.999328
	2.481585	0.184216	152.0697	154.0909	0.999727
	3.194002	0.067657	184.337	180	0.999362
	2.265894	0.155559	121.6212	123.0909	0.999542
	1.49348	0.45623	120.7826	114	0.999729
	1.877372	0.290352	199.7668	156	0.999215
	1.900708	0.238834	140.4435	129.25	0.99941
	3.037263	0.082603	154.7722	162.3333	0.999421
	2.926961	0.135728	175.7049	181	0.99951
	0.760592	0.49918	69.66579	71.85714	0.999469
	2.125427	0.253716	111.9171	100.4286	0.999513
	2.829032	0.141915	157.9455	154.3529	0.999506
	2.662174	0.114008	108.0149	106.8	0.99962
	2.280656	0.152932	92.34455	90.07692	0.999732
	2.841214	0.105753	113.1172	113.375	0.999663
	2.261122	0.15763	133.353	129.5	0.999484
	2.972117	0.091008	142.4623	147.125	0.999604

	1.576963	0.368314	84.80922	91.25	0.999453
	2.183189	0.236562	133.4352	137	0.999629
	3.436292	0.072627	171.2364	170.8333	0.999547
	2.254668	0.276006	163.0868	175.9091	0.999304
	2.792343	0.106499	191.0877	156.4	0.99956
	1.707424	0.420638	144.465	140.7692	0.999667
	2.858522	0.125387	156.7944	153.8125	0.999519
	2.963688	0.082889	146.8816	141.1053	0.999319
Moderately differentiatedly CRC	2.785381	0.107869	165.3865	160	0.999242
	1.711635	0.395193	110.8291	115.4286	0.99952
	3.009137	0.092347	171.4241	176.0909	0.999389
	2.776051	0.099615	108.1635	110.6667	0.99965
	1.633266	0.246741	67.08096	63.14286	0.999689
	2.551378	0.123871	96.29218	94.5	0.999707
	2.015028	0.230553	174.7373	140.25	0.999371
	2.666632	0.131665	157.4548	151	0.999492
	2.511357	0.143282	118.4199	116.75	0.999619
	2.401971	0.14657	108.6103	112	0.999487
	2.514941	0.172571	141.5396	137.4	0.999366
	2.505452	0.128806	101.6156	101	0.999624
	2.666119	0.120614	116.4628	102	0.999579
	2.144511	0.198178	92.02908	81	0.999648
	1.312191	0.462546	87.95165	156.5	0.99956
	2.128083	0.232391	91.68119	92.375	0.999616
	2.846306	0.111191	161.066	162.25	0.999482
	2.119695	0.348862	167.5754	163.6471	0.999395
	2.721433	0.13318	154.9042	158.6667	0.999444
	1.118497	0.480141	90.84403	99.5	0.999712
	2.451397	0.136384	99.65844	97.66667	0.999738
	2.843911	0.093873	197.8135	176	0.99912
	2.222542	0.171031	98.66164	97.42857	0.999732
	1.128816	0.450607	68.04203	43.42857	0.997708
	2.578868	0.121716	137.3489	135.2308	0.999646
	3.080095	0.076739	153.9357	177	0.999502
	1.183892	0.471431	94.65556	105	0.999538
	2.340316	0.203954	144.5664	139.3333	0.999873
	2.30352	0.164816	112.2981	110.0909	0.999405
	1.638105	0.280473	72.00454	73	0.999654
	2.703462	0.097397	116.9084	113.75	0.999561
	1.617566	0.419989	94.14047	100.6667	0.999582
	3.15549	0.065625	132.6451	131	0.999505
	2.073504	0.241642	114.5173	118.1111	0.999465
	2.051959	0.189331	146.0575	116.1667	0.999568
	2.398879	0.142676	111.9167	99.11111	0.999605
	2.221414	0.171514	109.9843	98	0.999524
	2.810905	0.13965	142.0243	132.875	0.999486
	2.312845	0.182147	145.8083	126.3333	0.999729
	2.78027	0.145521	108.4371	106.8	0.999804
	2.927401	0.087346	190.6137	206	0.99933
	2.544366	0.121962	127.5788	137.1429	0.99951
	2.376233	0.146843	131.527	122.9375	0.999653
	2.300641	0.146099	179.919	124.1	0.999596
	2.834784	0.099774	178.6538	156.8333	0.999399
	2.318734	0.183498	118.4143	121.75	0.999495
	3.113018	0.096144	169.5643	169	0.999499
	2.340293	0.17038	95.90058	93.125	0.999653
	3.348556	0.056398	171.7796	172.7692	0.999518
	1.511105	0.294702	105.5842	98.25	0.999429

2.925938	0.087517	160.8241	198.4286	0.999235
3.197	0.076666	183.9631	178.4	0.999425
2.829954	0.09788	121.5372	125	0.999617
1.990624	0.230046	160.3148	152.625	0.999427
2.15901	0.214513	130.2271	127.25	0.99933
3.090899	0.065568	112.5109	112.125	0.999625
2.066738	0.212964	98.66764	98.8	0.999887
2.647792	0.112387	127.6852	131.6667	0.999489
1.706139	0.401258	155.8177	172	0.999293
2.583677	0.179126	133.4707	161.6	0.99963
3.07096	0.075558	143.4045	147	0.999472
2.051309	0.263868	124.4361	126.3333	0.999457
2.724752	0.103414	129.6168	130.2727	0.999517
2.594693	0.131503	133.8183	127.3333	0.999703
3.273925	0.063378	166.9268	177.25	0.999573
2.556045	0.152562	130.5562	132.125	0.999828
1.28365	0.457204	93.27248	93.11111	0.99981
2.080875	0.163531	151.1235	96	0.99951
1.972435	0.2596	123.8022	105.25	0.999626
2.81868	0.081786	93.11082	97	0.999692
3.166731	0.110717	158.694	156.9286	0.999478
0.59349	0.805717	80.04018	78.125	0.999619
2.28695	0.148947	179.3596	127.625	0.999295
2.467899	0.152671	89.32691	93	0.999734
2.310843	0.187561	141.0402	140.25	0.999774
3.070109	0.085861	182.6068	185.0769	0.999364
1.953067	0.200922	169.2735	167.5	0.999319
2.137441	0.205251	110.7064	124.6	0.999613
2.239826	0.198468	131.5253	127	0.999534
1.790539	0.25546	216.5736	187.5556	0.999057
2.426181	0.173531	148.7675	155.2727	0.999666
1.570647	0.338842	143.4033	129.1	0.999681
3.190882	0.077877	155.5498	156.3636	0.99961
3.098567	0.062919	144.2672	150.6667	0.999502
1.55962	0.424785	109.5232	113.125	0.999706
0.502653	0.805172	109.5555	70.11111	0.999481
3.033638	0.085692	186.8848	192.8	0.999608
2.756372	0.122002	134.6276	133.2727	0.999681
1.936378	0.231637	88.19737	93.6	0.999788
2.537718	0.120751	87.61941	85.875	0.999769
2.580272	0.182846	104.5691	115.75	0.999602
2.365112	0.246064	143.9245	146.3	0.999514
0.949499	0.633813	116.0988	108.5	0.999697
1.766843	0.254234	78.61745	80.11111	0.999457
1.853691	0.29323	196.9548	219.1667	0.999245
3.143827	0.080658	158.391	170.625	0.999427
2.555221	0.145433	104.243	104.3333	0.999545
3.104423	0.085833	141.3412	141.2727	0.999646
2.4556	0.139482	87.84214	87.66667	0.999639
3.516777	0.05149	206.7833	201.5	0.999282
1.530805	0.33956	126.8865	106.6667	0.99956
1.602616	0.425123	107.4554	112.8571	0.999744
2.317943	0.192751	115.7486	118.1	0.999573
1.737243	0.268284	92.47177	87.57143	0.999702
3.40088	0.066407	195.1844	204.9091	0.999175
2.715591	0.132784	160.1186	183.375	0.999358
2.444364	0.159587	120.7282	106.1	0.999659
1.651144	0.333235	149.9866	110.8333	0.999188

	2.520011	0.15582	130.5946	127	0.99948
	3.03349	0.088238	170.6864	180	0.999401
	2.55748	0.14219	117.8496	115.5833	0.999686
	2.552579	0.130225	150.3176	145.6471	0.99945
	1.981456	0.264886	90.75826	93.6	0.999697
	2.497677	0.222274	155.5359	157.2727	0.999581
	2.027459	0.238589	123.3045	106.75	0.999709
	2.384538	0.244305	131.1335	117.125	0.9995
	2.67311	0.148675	170.7231	164.6471	0.99933
	2.864508	0.082329	113.3914	107	0.999599
	2.645259	0.123786	121.1925	118.5455	0.999519
	2.494809	0.177849	109.2205	105.0769	0.999725
	2.718432	0.101089	103.409	99.07692	0.999688
	3.403813	0.055661	177.9283	194	0.999169
	2.252725	0.288104	195.1235	192.8	0.999128
	3.257295	0.071746	173.5177	179	0.9995
Poorly differentiatedly CRC	2.34074	0.167457	263.9093	188.5	0.999001
	2.00442	0.223135	113.8078	96.375	0.999606
	1.914305	0.257345	111.9688	114	0.999567
	3.453555	0.051277	189.4367	199.1	0.999468
	2.534391	0.131098	102.0447	103.1429	0.999725
	2.94177	0.136698	148.2871	144.6667	0.999503
	2.010935	0.313033	120.5903	135.5	0.999532
	2.595697	0.121702	146.6494	130.6667	0.999415
	2.732514	0.115269	140.8367	140	0.999554
	3.352841	0.067742	178.7673	185.2727	0.999456
	2.204694	0.274328	117.9583	124.5	0.999757
	1.222935	0.540224	111.9486	95.27273	0.999561
	2.492463	0.127634	113.1729	119.75	0.999497
	3.33457	0.060184	152.9956	155.25	0.999441
	2.221143	0.248198	181.2879	188.5	0.999312
	2.5803	0.159501	148.5111	148	0.999727
	2.226729	0.22563	117.59	118.3636	0.99944
	2.173456	0.250036	137.7312	144.3333	0.999345
	2.780491	0.093148	183.3702	177	0.99939
	2.258466	0.212148	119.8346	118.5455	0.99953
	2.529632	0.158195	138.6544	138.2143	0.999248
	2.823954	0.099992	127.9789	123.1	0.999697
	1.023453	0.614537	75.68182	76	0.999627
	1.054803	0.539909	133.8052	108.8571	0.99922
	2.331175	0.2339	147.015	145.8333	0.99948
	2.075808	0.161846	94.65856	91.33333	0.999529
	1.509168	0.453268	145.5384	158.9091	0.999408
	2.491721	0.149366	137.9394	142	0.999594
	2.65696	0.119787	142.6823	138.875	0.99963
	3.009325	0.077095	114.6873	117	0.999584
	2.700598	0.104321	129.3346	140.5	0.999218
	2.432697	0.168192	156.7509	148.5	0.999688
	3.057027	0.094621	144.951	144.5455	0.99962
	2.475089	0.180408	132.0793	129.3333	0.999544
	3.264483	0.069195	175.7846	176.4	0.999242
	2.30491	0.15459	124.9935	125.25	0.999536
	0.298037	0.917041	130.6853	97.5	0.999346
	1.72636	0.431241	111.4453	122.5	0.999456
	3.105634	0.067413	148.63	139.25	0.999416
	3.067071	0.073307	156.314	152.25	0.999667
	2.519613	0.109495	113.9925	108.0769	0.999859
	2.897591	0.108432	166.8999	175	0.999339

2.661297	0.111019	112.6368	114	0.999767
2.862444	0.082136	123.1483	117.2308	0.999585
1.895715	0.232105	92.08474	95.125	0.99977
1.969438	0.250359	85.86598	88	0.999718
2.906228	0.092313	156.3766	190	0.999411
2.830096	0.1349	164.6905	163.0833	0.999332
1.835666	0.277419	109.5808	117.625	0.999521
2.608062	0.103347	100.5398	105.6667	0.999587
2.483994	0.150703	199.6264	142.2143	0.999142
1.728867	0.288435	145.2716	152	0.999642
2.715058	0.140543	180.9864	162.0833	0.999347
3.157605	0.078459	138.8544	139.1111	0.999698
1.476936	0.354955	91.30504	93	0.999551
2.398411	0.155219	112.3615	107.5714	0.999861
2.307666	0.147278	86.51021	72.16667	0.999716
2.359221	0.1705	121.9932	111.0714	0.99941
1.210758	0.573837	112.2934	114.3	0.999529
1.831288	0.214791	97.25627	100.375	0.999738
0.928745	0.668141	89.14049	88.8	0.999664
1.385656	0.485584	106.7339	117.8571	0.999355
